# Assessing the consistency of iPSC and animal models in cystic fibrosis modelling: A meta-analysis

**DOI:** 10.1371/journal.pone.0272091

**Published:** 2022-08-09

**Authors:** Toqa Darwish, Azhar Al-Khulaifi, Menatalla Ali, Rana Mowafy, Abdelilah Arredouani, Suhail A. Doi, Mohamed M. Emara

**Affiliations:** 1 Basic Medical Sciences Department, College of Medicine, Qatar University, Doha, Qatar; 2 Diabetes Center, Qatar Biomedical Research Institute (QBRI), Hamad Bin Khalifa University (HBKU), Education City, Qatar Foundation, Doha, Qatar; 3 Department of Population Medicine, College of Medicine, QU Health, Qatar University, Doha, Qatar; Sapienza University of Rome, ITALY

## Abstract

**Introduction:**

Cystic fibrosis (CF) is a hereditary autosomal recessive disorder caused by a range of mutations in the CF Transmembrane Conductance Regulator (CFTR) gene. This gene encodes the CFTR protein, which acts as a chloride channel activated by cyclic AMP (cAMP). This meta-analysis aimed to compare the responsiveness of induced pluripotent stem cells (iPSCs) to cAMP analogues to that of commonly used animal models.

**Methods:**

Databases searched included PubMed, Scopus, and Medline from inception to January 2020. A total of 8 and 3 studies, respectively, for animal models and iPSCs, were analyzed. Studies were extracted for investigating cAMP-stimulated anion transport by measuring the short circuit current (*I*_sc_) of chloride channels in different animal models and iPSC systems We utilized an inverse variance heterogeneity model for synthesis.

**Results:**

Our analysis showed considerable heterogeneity in the mean *I*_sc_ value in both animal models and iPSCs studies (compared to their WT counterparts), and both suffer from variable responsiveness based on the nature of the underlying model. There was no clear advantage of one over the other.

**Conclusions:**

Studies on both animal and iPSCs models generated considerable heterogeneity. Given the potential of iPSC-derived models to study different diseases, we recommend paying more attention to developing reproducible models of iPSC as it has potential if adequately developed.

## 1 Introduction

Cystic fibrosis (CF) is the most widespread monogenic fateful respiratory disease. It affects over 89,000 individuals worldwide, and there are about 1000 new cases each year [1]. It is an autosomal recessive disorder resulting from loss of function mutations in the cystic fibrosis transmembrane conductance regulator (CFTR) gene that prevents the expression of normal function of the CFTR protein, a regulated chloride channel located in the cell membrane. In CF, thick and sticky mucus forms in the lungs, impeding breathing and providing a nutritious environment for pathogens to survive, and eventually may lead to premature respiratory failure [2]. CF affects multiple organ systems other than the lungs and can result in complications in the liver, pancreas, and small bowel [3].

Over the last two decades, considerable advances have been achieved in understanding the molecular and cellular mechanisms involved in CF etiology. Consequently, substantial improvement in the care and management, and ultimately extension in the life expectancy of CF patients, has been achieved [4]. However, given the lack of a cure for CF, the disease can still interfere with the quality of life and eventually cause a significant shortening of life expectancy in many cases. Therefore, the development of new therapeutic approaches is of paramount clinical importance.

To develop a new therapeutic approach to human disease, it is essential to obtain substantial knowledge of its underlying cellular and molecular mechanisms. This step is typically carried out using disease-modeling animals. Animal models are also crucial for the clinical evaluation of new medications before being administered to humans. In previous studies, animal models have been of great use in elucidating specific mechanisms involved in CF pathophysiology, and the development of new therapies [5]. However, research has revealed the difficulty, and sometimes failure, of animal models to mimic human tissues or organs. For example, in many cases, mouse models fail to resemble the phenotypic features of CF patients [6]. Hence, animal models are not always reliable for human pathologies such as CF.

The generation of induced pluripotent stem cells (iPSC) from adult somatic cells is likely to revolutionize the production of in vitro disease models [7, 8]. It has been possible to differentiate iPSCs derived from patients with inherited diseases into specific cell types that can recapitulate some of the key pathological changes seen in diseases such as amyotrophic lateral sclerosis (ALS) [9], spinal muscular atrophy (SMA) [10], and Parkinson’s disease (PD) [11]. Thus, the iPSC technology is offering an unprecedented opportunity to gain insights into disease mechanisms and to search for new drugs using human disease-specific cell lines [12]. Although the iPSC-based therapy is still in its infancy, the ability to model human disease in vitro could be made immediately valuable with patient-specific iPSCs. In this regard, thanks to iPSC, significant progress in the understanding of Alzheimer’s disease (AD) has been made in recent years, opening up new avenues for combating AD [13]. Other approaches that utilized iPSCs in modeling CF pathogenesis are conditionally reprogrammed cell (CRC) [14], human nasal epithelial stem cells, based on dual SMAD inhibition [15], and Human primary airway epithelial cell (HAE) models [16]. Indeed, CRC shows promising results similar to iPSCs in modeling the disease, where it has previously been demonstrated that CRC stem-like cells expanded from CF patients’ lung tissue could generate a large number of airway epithelial stem cells [14]. This created the so-called CF-CRC model that truly mimics patients’ respiratory tissues. Importantly, this patient-specific model was used to test for various CF genotypes’ drug responses, which paved the way for clinical application. Similarly, dual SMAD inhibition showed an efficient expansion of primary epithelial basal cell populations to produce functional airway epithelium that shows a physiological response to different CFTR drugs, such as CFTR modulators [15].

To date, very little research has been conducted to model CF disease using human iPSC models, either for understanding its pathophysiology or testing drug efficacy. Nevertheless, the number of diseases that were successfully mimicked using iPSC is progressively increasing [17], indicating the growing utility of iPSCs for studying disease development, investigating pathophysiology, and testing therapeutic agents. The unknown is which of the two models (animal/iPSC) will be the best at mimicking the disease and thus useful for testing target treatments. In the case of CF, many studies have reported chloride channel function in mutated cell lines in comparison to WT cells under cAMP analogue stimulation and this difference has been used as a proxy to assess models with the greatest potential for assessing therapeutic targets (assuming that consistency of responsiveness to c-AMP analogues across studies suggests a good model).

When describing any new epithelial animal/culture models, it is critical to include information on whether the cell models exhibit vectorial transport of chloride ions, which is the primary function of epithelia and can be assessed through the measurement of transepithelial short-circuit current (I_sc_). This meta-analysis was therefore undertaken to compare the ion transport function of epithelia from animal models and CF-patient-derived iPSC models under cAMP analogues. The response will be defined by the difference in short circuit current between model-derived cells and WT cells normalized to the epithelial surface area. This difference in responsiveness was compared across studies for each model (animal / iPSC).

## 2 Materials and methods

### 2.1 Search strategy

All studies using animal models or iPSCs to investigate different pathologies of CF were searched in the PubMed, Scopus, and Medline databases. The PubMed search utilized the Medical Subjective Heading (MeSH) terms “Cystic Fibrosis”, “Animal Models”, and “Induced Pluripotent Stem Cell(s).” The other databases were searched using equivalent terms, and all searches were from inception to January 2020. In addition, bibliographies of past meta-analyses and systematic reviews on animal models were revised for additional studies related to the animal or iPSC models in CF.

### 2.2 Inclusion and exclusion criteria and data extraction

The inclusion criteria for our studies were those that: i) compared cystic fibrosis models (animal or iPSC) to WT cells; ii) used cAMP stimulation through agents such as, but not limited to, Forskolin; iii) included adequate data, including mean and standard deviation (SD) of short circuit current of chloride channels for each group (stimulated or non-stimulated); v) were published in peer-reviewed journals with full text available in English.

Studies were excluded if they were editorials, case reports, news, letters, reviews, commentaries, or retracted articles. Further exclusion of eligible studies was due to duplicate studies or lack of data, such as standard deviations not being available.

Two authors extracted the studies independently with follow-up discussions to screen for duplicates and omit them. The data from each article was collected as follows: Authors’ names, date of publication, iPSCs model used, Animal Model used, breed and genotype, any electrophysiological measurement as mean and standard deviation, number of trials, and different types and concentrations of stimulatory or inhibitory agents used. Studies reporting standard error of the mean (SEM) instead of SD were extracted, and the SEM was converted to SD. For studies where data was presented as charts, a graph digitizer software, WebPlotDigitizer, was utilized [18]. We also contacted authors for numerical data.

### 2.3 Quality assessment

The quality of studies extracted was evaluated using SYRCLE’s risk of bias tool for animal studies, a 10-item quality scale [19]. The scale consisted of the following domains: i) sequence generation, ii) baseline characteristics, iii) allocation concealment, iv) random housing, v) blinding (in terms of reducing performance bias), vi) outcome assessment, vii) blinding (in terms of reducing detection bias), vii) incomplete outcome data assessment, ix) selective outcome reporting, x) other sources of bias.

**Fig 1:**
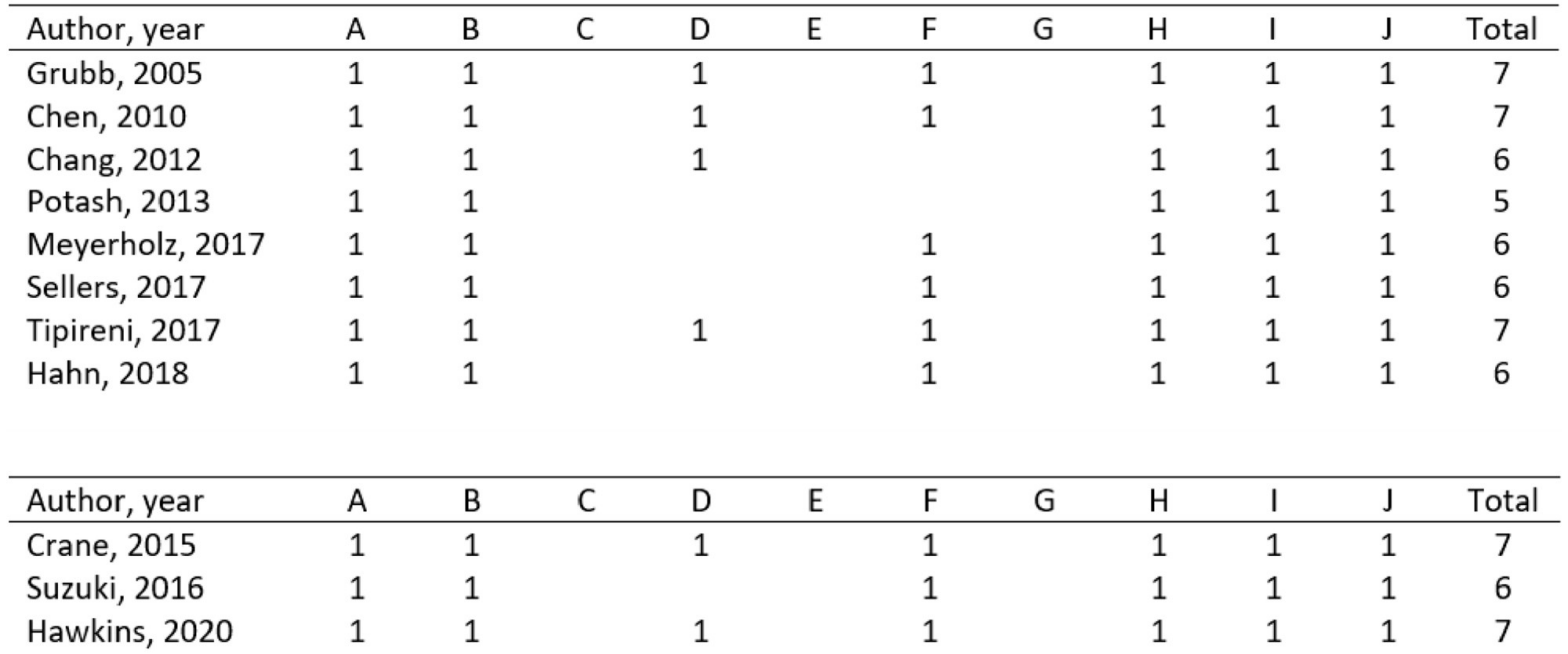
Risk of Bias Assessment of included studies, Top Panel: Animal Models Studies, Bottom Panel: iPSCs Studies. A: Sequence generation, B: Baseline characteristics, C: Allocation concealment, D: Random housing, E: Blinding (in terms of reducing performance bias), F: Random outcome assessment, G: Blinding (in terms of reducing detection bias), H: Incomplete outcome data assessment, I: Selective outcome reporting, J: Other sources of bias. References: Sellers [27]; Meyerholz [26]; Hahn [25]; Grubb [31]; Potash [33]; Chang [34]; Chen [35]; Tipirneni [32]; Suzuki [28]; Hawkins [30]; Crane [29].

To calculate the number of safeguards implemented in each study, we assigned one point for each implemented safeguard (scale item). The study counts were then converted to a relative rank by dividing the total count for each study by the highest observed total count in the list of studies [20]. The range of ranks for animal model studies was 0.71–1, while the range of ranks for iPSC model studies was 0.86–1 (Fig 1). Most studies lost internal validity safeguards because of a lack of experimental design and thus a lack of randomization and/or blinding. This could be because this type of research does not lend itself well to an experimental epidemiological design.

### 2.4 Statistical analysis

This study compares mutated (animal or iPSC models) versus WT CFTR chloride channels under cAMP analogue stimulation. The cAMP analogues used in these studies include Forskolin, IBMX and FGF-10. Because they all acted as cAMP analogues, all the agents used were considered similar stimulants. The outcome of interest was the function of the mutated channel relative to WT function under cAMP analogue stimulation in either animal models or iPSCs. A good model would be indicated by the consistency of the difference between stimulated chloride channel function in the WT and mutated cell types across studies. The channel function was measured by the Ussing Chamber system (UCS) through the recording of electrophysiological characteristics of tissue samples (from animal models) and cell culture (from iPSCs models). The characteristic measure was the short circuit current (I_sc_) which is the transepithelial current needed to clamp the transepithelial voltage to zero and is equal to the sum of the active ion transport processes operating across the epithelium [21]. This current is normalized to epithelial surface area and measured in μA/cm^2^ and reported as mean and standard deviation. As such, the weighted mean difference (mutated vs WT) in short circuit current levels was used as the effect size in this study. Both a larger difference as well as a consistent difference (i.e., lack of heterogeneity) between WT and mutated groups represents a better model for CF.

A forest plot was generated to illustrate the weighted mean differences (WMDs) and confidence intervals (CI) of each study. Pre-specified subgroup analyses were conducted for animal model studies at pre-defined subgroups based on animal and tissue types, cAMP analogue types, and concentrations used in each study. The subgroup analyses mentioned above were undertaken to help determine whether variations in the methods of different animal model studies had a significant role in heterogeneity between these studies (high I^2^ values). As the iPSC studies used the same model and followed similar experimental methodologies, we did not perform a subgroup analysis for these studies.

An inverse variance heterogeneity (IVhet) model was utilized for meta-analysis, and heterogeneity was quantified using the I squared (I^2^) statistic. The I^2^ statistic estimates the percentage of variation across studies that is due to heterogeneity rather than chance [22, 23]. Heterogeneity can be caused by chance, which is expected and normal, or by systematic error due to a variety of factors. An I^2^ value below 50% indicates low or minimal heterogeneity, whereas an I^2^ value of 50% or higher usually indicates the presence of moderate to high heterogeneity across the studies. Publication bias was examined using the Doi plot and the Luis Furuya-Kanamori (LFK) index values in groups with at least five studies. All statistical analyses were performed using the MetaXL add-in on Microsoft Excel and Stata statistical software, version SE 16 (Stata Corporation, Texas, USA).

## 3 Results

### 3.1 Study selection process

**Fig 2:**
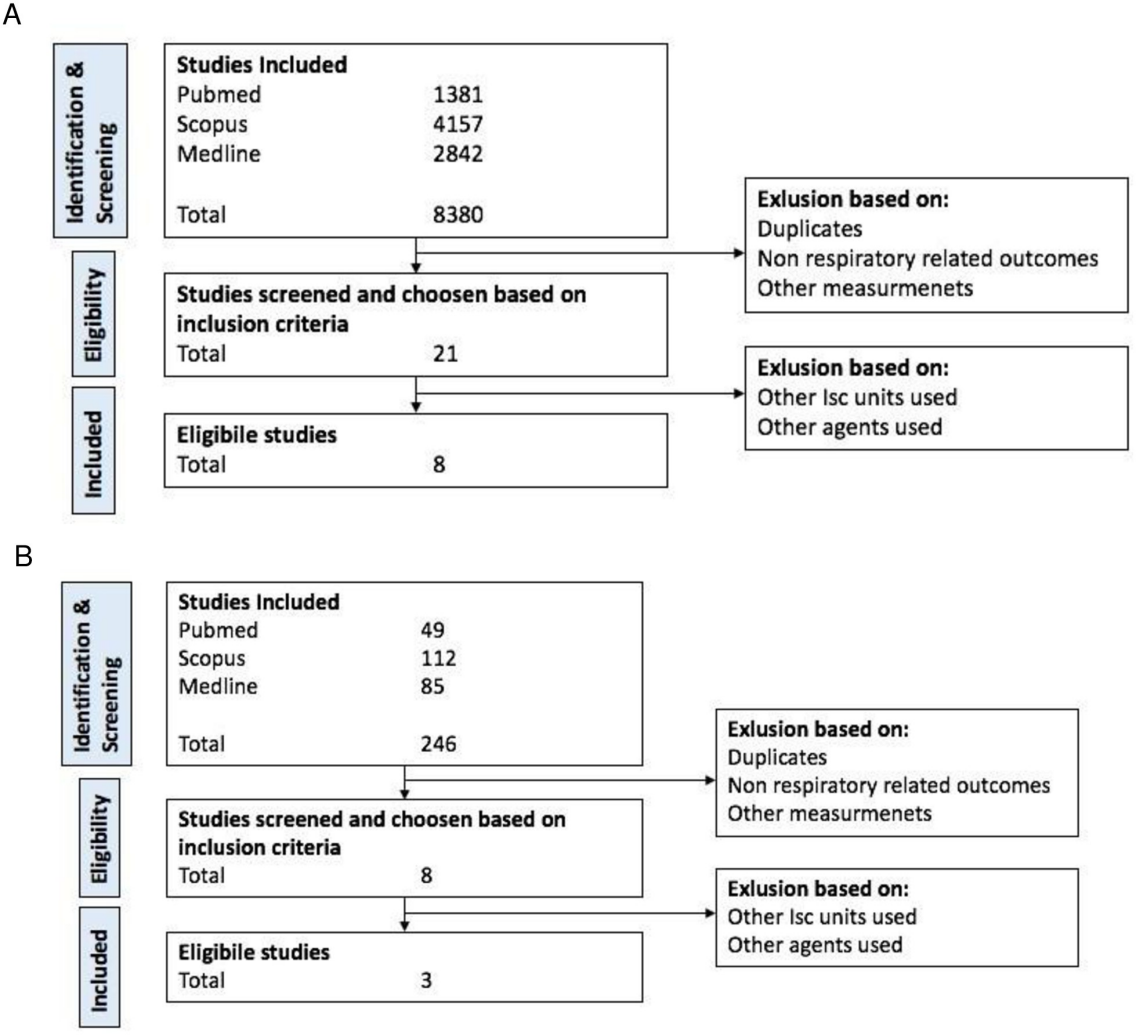
Study selection process of Animal Models studies (A) and iPSCs studies (B).

Fig 2 depicts the Quorum flow diagram of our study selection process. Using the keywords “animal models”, “iPSCs” and “cystic fibrosis”, a total of 8380 studies were selected for the animal models group and 246 studies for the iPSCs group. These were retrieved from three databases: PubMed, Scopus, and Medline. Following the screening stage, 21 studies were eligible for inclusion in the animal models group and 8 for inclusion in the iPSCs group based on the titles and abstracts of the studies. In the end, our inclusion criteria were met by 8 animal model studies and 3 iPSCs studies.

### 3.2 Description of included studies

Table 1 displays the detailed characteristics of the studies included in our meta-analysis. In the animal CF model group, four studies utilized pig models (50%), three used murine models, and one used a ferret model. These three animals are the most commonly used in CF research [5, 24], and thus can be considered a representative selection. All studies used the UCS to measure the I_sc_. Out of the 8 animal model studies, 5 used Forskolin and IBMX solutions as cAMP analogues (63%). Two of these studies used additional agents, including NKCC1-inhibitor bumetanide [25], and FGF-10 [26]. Out of the remaining three studies, two used Forskolin as the only analogue, and the last one used Amiloride and PGE2 [27].

**Table 1:** Characteristics of included studies.

Author Name	Year	Model	Specie	Tissue	Solution	Concentration	Measurement	Method
Sellers [27]	2017	Animal	Ferret	Trachea	Amiloride + PG2	10 μM amiloride/ 1 μM PGE2	Short circuit current: Chloride and Bicarbonate secretion	Ussing Chamber
Meyerholz [26]	2017	Animal	Pig	Trachea	Forskolin, IBMX, FGF-10	100 ng/ml FGF-10 / 100 μM IBMX / 10 μM Forskolin	Short circuit current	-
Hahn [25]	2018	Animal	Murine	Trachea + Bronchi	IBMX/FSK NKCC1-inhibitor bumetanide	100/1 μM 100 μM	Short circuit current: CFTR dependent chloride secretion	Ussing Chamber
Grubb [31]	2005	Animal	Murine	Nasal Epithelia	Forskolin	10–5 M	Short circuit current: Chloride Conductance	Ussing Chamber
Potash [33]	2013	Animal	Pig	Sinus Epithelia	Forskolin/ IBMX	F: 10 mmol/l + IBMX: 100 mmol/l	Short circuit current	Ussing Chamber
Chang [34]	2012	Animal	Pig	Sinus Epithelia	Forskolin/ IBMX	F: 10 mM + IBMX: 100 mM	Short circuit current	Ussing Chamber
Chen [35]	2010	Animal	Pig	Trachea + Nasal	Forskolin/ IBMX	F: 10 μM + IBMX: 100 μM	Short circuit current: Chloride and Bicarbonate secretion	Ussing Chamber
Tipirneni [32]	2017	Animal	Murine	Nasal Epithelia	Forskolin	20 μM	Short circuit currents: Anion Transport	Ussing Chamber
Suzuki [28]	2016	iPSC	Human	Fibroblast (F508del/F508del)	Amiloride	20 μmol/L	Short circuit current: Chloride current	Ussing Chamber
Hawkins [30]	2020	iPSC	Human	Putative basal cells (iBC)	Amiloride + Forskolin	100 μM / 10 μM	Short circuit current Transepithelial chloride transport	-
Crane [29]	2015	iPSC	Human	Fibroblast (ΔF508/ΔI507)	Forskolin	20 μM	Short circuit current	Ussing Chamber

Abbreviations:

a. iPSC: induced pluripotent stem cells

b. PGE2: Prostaglandin E2

c. FGF-10: Fibroblast growth factor 10

d. IBMX: 3-isobutyl-1-methylxanthine

All studies included in the iPSCs group utilized human CF fibroblasts and used UCS to measure the I_sc_. One study used Amiloride only [28], another used Forskolin only [29], and the remaining study used both agents [30]. Whilst one of the iPSCs studies did not mention the genotype of the CF fibroblasts, one study reported the use of heterozygous fibroblasts with the mutation DF508/DI507 [29], and the other used homozygous fibroblasts with the DF508/DF508 mutation [28].

### 3.3 Meta-analysis

#### 3.3.1 Animal models

**Fig 3:**
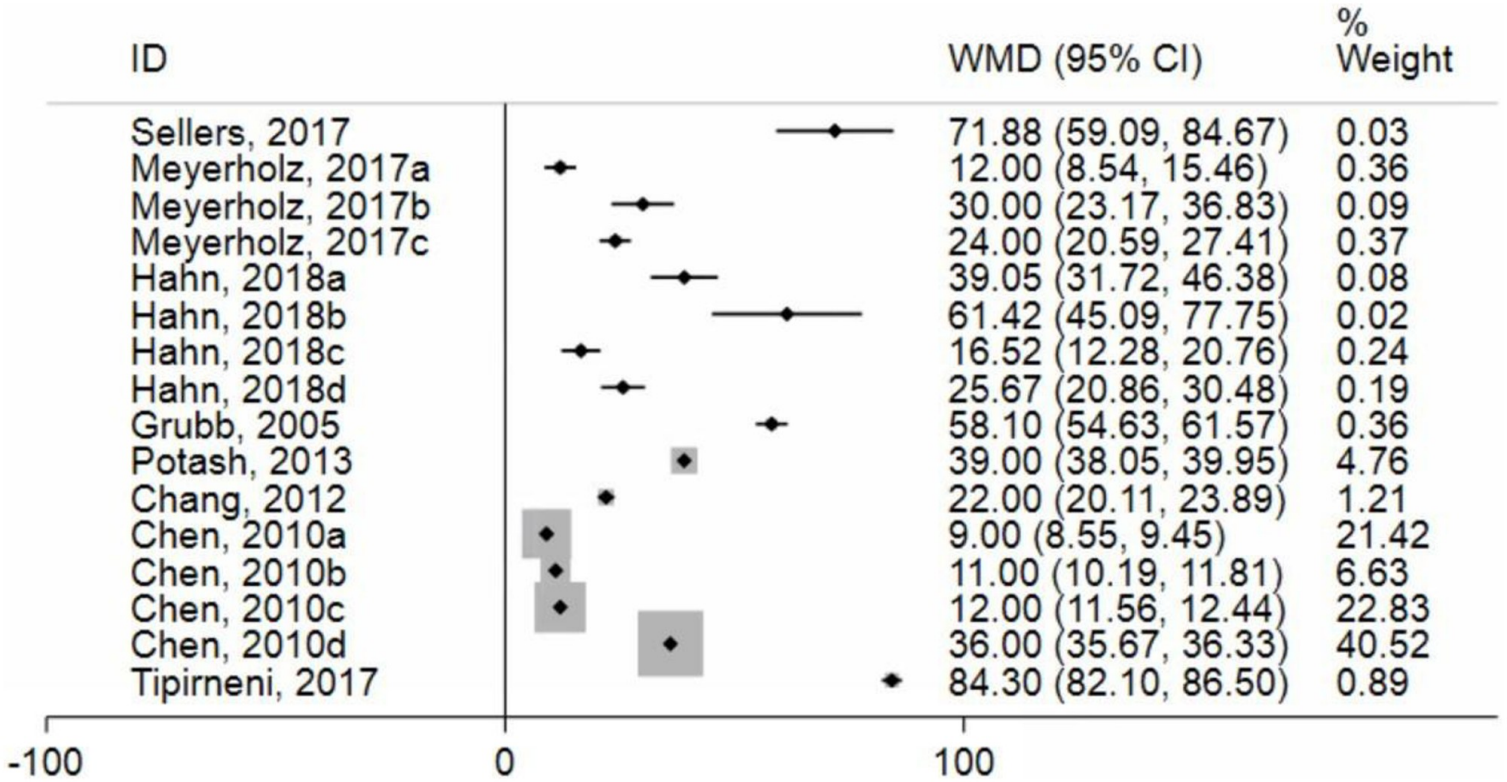
Forest plot of included animal models studies. References: Sellers [27]; Meyerholz [26]; Hahn [25]; Grubb [31]; Potash [33]; Chang [34]; Chen [35]; Tipirneni [32]; Suzuki [28]; Hawkins [30]; Crane [29].

**Fig 4:**
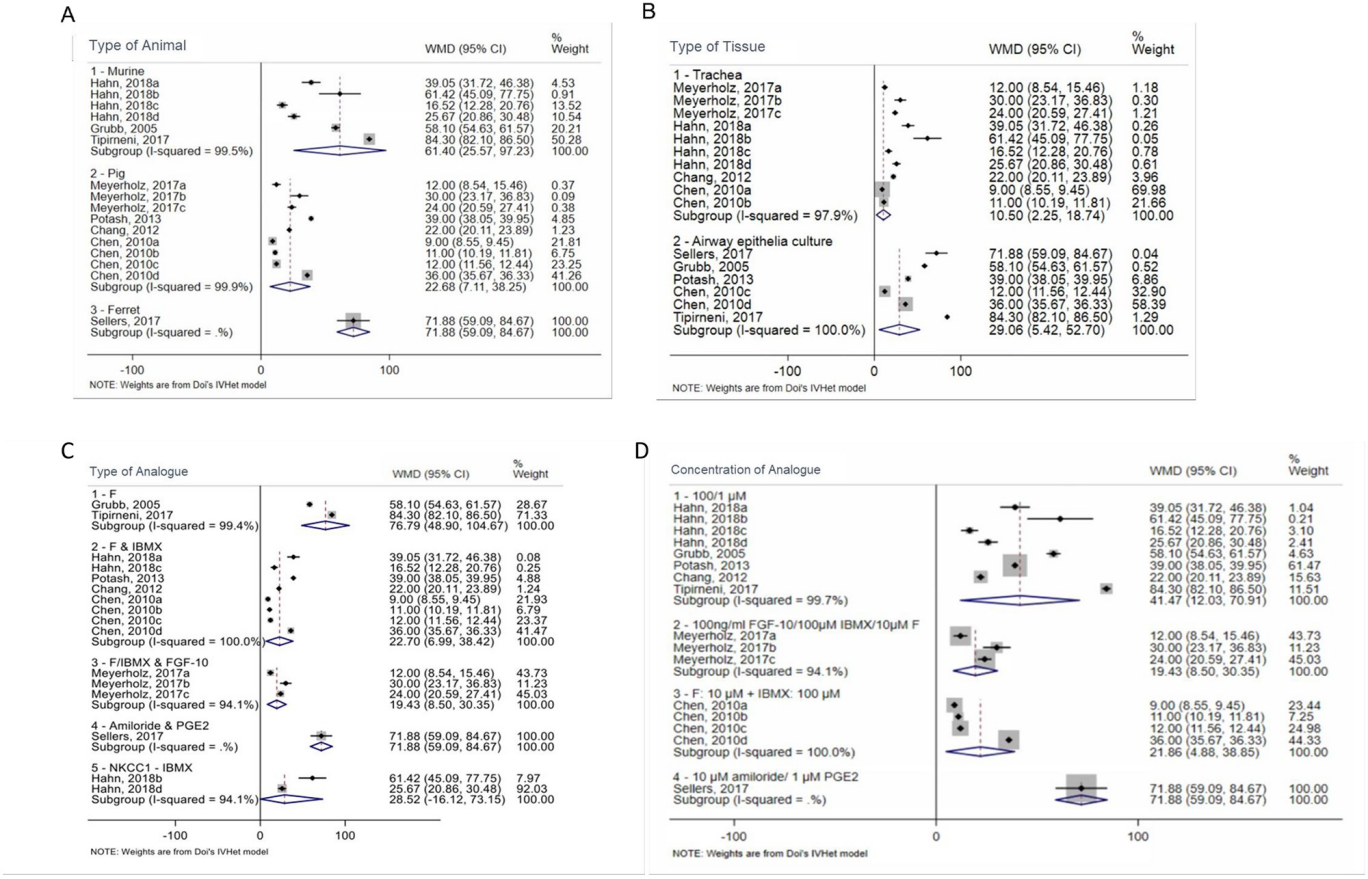
Subgroup analysis based on Animal Type (A), Tissue Type (B), Analogue type used (C) and Concentration of analogue used (D). F: Forskolin, IBMX: 3-isobutyl-1-methylxanthine, FGF-10: Fibroblast Growth Factor 10, PGE2: Prostaglandin E2, NKCC1: Na-K-Cl cotransporter protein. References: Sellers [27]; Meyerholz [26]; Hahn [25]; Grubb [31]; Potash [33]; Chang [34]; Chen [35]; Tipirneni [32]; Suzuki [28]; Hawkins [30]; Crane [29].

Overall, there was significant heterogeneity in animal model studies, as evidenced by an I^2^ of 99.9% (Fig 3). The studies were divided into three groups based on the type of animal used: mice (group 1), pigs (group 2), and ferrets (group 3). There was a total of three studies in the mouse group, one of which included four separate experiments, making a total number of six data points for analysis [25, 31, 32]. The pig group included a total of 4 studies, with a total of 9 data points [26, 33, 35]. The ferret group included one study with one data point [27]. The WMDs of I_sc_ were 61.4 μA/cm^2^ (95% CI 25.57–97.23) for the murine group, 22.68 μA/cm^2^ (95% CI 7.11–38.25) for the pig group, and 71.88 μA/cm^2^ (95% CI 59.09–84.67) for the ferret group (Fig 4A). A test of interaction indicated a subgroup effect (df = 2; p < 0.001).

The I^2^ value was high in both the murine (99.5%) and pig (99.9%) subgroups (Fig 4A). In contrast, in the ferret group, there was only one study. Therefore, even after subgrouping studies based on the use of the same type of animal model, results remained significantly heterogeneous.

*3*.*3*.*1*.*1 Tissue type subgroup analysis*. The next variable we looked at was the type of tissue used in each study. We divided the studies into two categories: those that used excised tracheal or bronchial tissue (group 1) and those that used cultured airway tissue (group 2). A total of 10 data points were identified for group 1, retrieved from 4 studies, and 6 data points for group 2 from 5 studies. The WMDs of Isc were 10.50 μA/cm2 (95% CI 2.25–18.74) for the tracheal tissue group and 29.06 μA/cm2 (95% CI 5.42–52.70) for the airway epithelia culture group (Fig 4B). A test of interaction did not indicate a subgroup effect (df = 1; p = 0.146). The I^2^ values in groups 1 and 2 were significantly higher, with 99.6% and 100% heterogeneity, respectively (Fig 4B).

*3*.*3*.*1*.*2 Analogue type and concentration subgroup analysis*. Further exhaustive analysis was done for each paper based on the types of analogues used and their concentrations. These analogues generate cAMP and were used to aid in determining the optimal function of the CFTR protein.

*3*.*3*.*1*.*2*.*1 Analogue type*. We conducted a sub-analysis for each type of analogue used in animal models. The results of the studies that used the same types of solutions were grouped together. The analogues were categorized as Forskolin (group 1), Forskolin and IBMX (group 2), Forskolin with IBMX and FGF-10 (group 3), amiloride and PGE2 (group 4), and lastly NKCC1- inhibitor bumetanide (group 5). The WMD of I_sc_ was 76.79 μA/cm^2^ (95% CI 48.90–104.67) for group 1, 22.70 μA/cm^2^ (95% CI 6.99–38.42) for group 2, 19.43 μA/cm^2^ (95% CI 8.50–30.35) for group 3, 71.88 μA/cm^2^ (95% CI 59.09–84.67) for group 4, and finally 28.5 μA/cm^2^ (95% CI -16.12–73.15) for group 5 (Fig 4C). A test of interaction indicated a subgroup effect (df = 4; p < 0.001).

The overall heterogeneity of the results from different types of analogues used in the studies had an I^2^ value of 99.9%. In group 1, the results from two studies were heterogeneous with an I^2^ value of 99.4% (Fig 4C). The results from group 2 were also heterogenous with an I^2^ value of 100% (Fig 4C). Group 3 contained only one study, Meyerholz et al. [26], but contained three different trials of the same animal and tissue type, and the results were also heterogenous with an I^2^ value of 94.1% (Fig 4C). Moreover, group 4 contained only one study, Sellers et al. [27]. Finally, group 5 contained one study, Hahn et al. [25], with two different tissue types and the results were heterogenous with an I^2^ value of 94.1% (Fig 4C).

*3*.*3*.*1*.*2*.*2 Concentration of analogues*. Analogues’ concentrations used were divided into three groups. Group 1 contained all the studies that used a concentration of 100 μM of cAMP analogue, their results were heterogenous with an I^2^ of 99.7%. Group 2 contained three trials from the same study, Meyerholz et al. [26], and used a solution concentration of 100 ng/ml FGF-10 + 100 μM IBMX + 10 μM Forskolin for the same animal and tissue, the results were heterogeneous with an I^2^ value of 94.1%. Group 3 contained four trials from the same study, Chen et al. [35], which used 10 μM Forskolin + 100 μM IBMX for the same animal type but different tissue types, their results were heterogenous with an I^2^ value of 100% (Fig 4D). Group 4 contained the concentration used for the study of Sellers et al. [27] study. The overall heterogeneity of the results from different analogue concentrations used in the papers had an I^2^ value of 99.9%.

The WMD of I_sc_ was 41.47 μA/cm2 (95% CI 12.03–70.9) for group 1, 19.43 μA/cm2 (95% CI 8.50–30.35) for group 2, 21.86 μA/cm2 (95% CI 4.88–38.85) for group 3, 71.88 μA/cm2 (95% CI 59.09–84.67) for group 4 (Fig 4D). A test of interaction indicated a subgroup effect (df = 3; p < 0.001).

#### 3.3.2 Induced pluripotent stem cells (iPSC)

**Fig 5:**
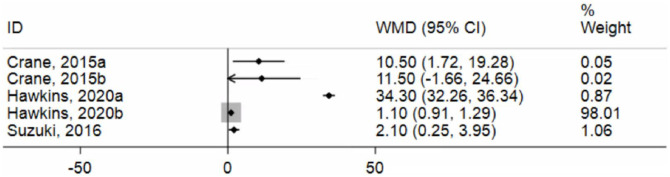
Forest plot of included IPSCs studies. References: Suzuki [28]; Hawkins [30]; Crane [29].

As mentioned above, three iPSC papers were included in our analysis, with a total of 5 data points. As the studies used the same model and followed similar experimental methodologies, we did not prespecify any subgroup analyses. The I^2^ value obtained for iPSCs studies was 99.6%, indicating significant heterogeneity like that demonstrated for animal models (Fig 5).

### 3.4 Publication bias

**Fig 6:**
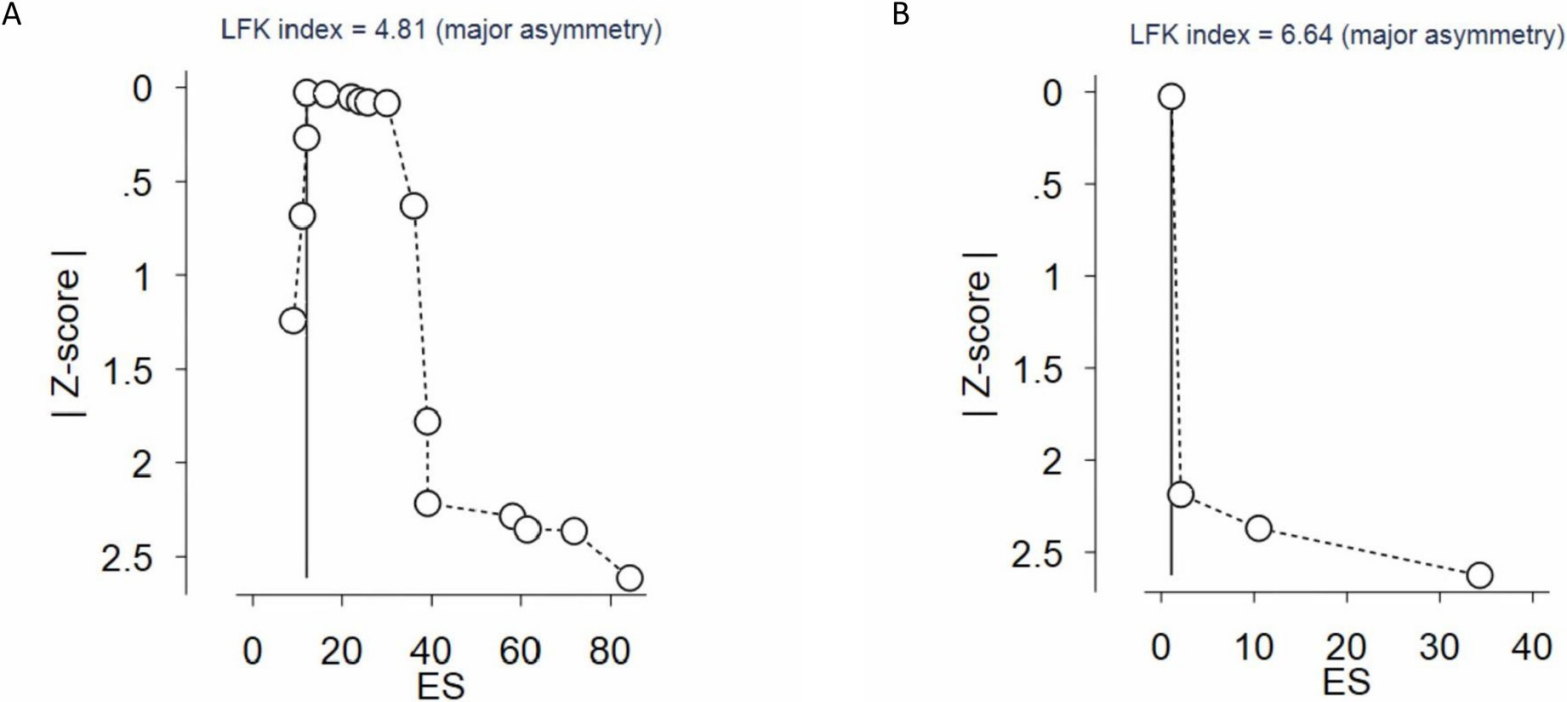
Doi Plots for Animal Models (A) and IPSCs (B).

For the identification of publication bias, we used the Doi plot and the LFK index to visually assess and quantify the degree of asymmetry. We found an overall LFK index value of 4.81, indicating significant positive asymmetry in both the animal and iPSCs models (Fig 6A & 6B). The LFK index was higher in the iPSC group, at 6.64. These findings imply that studies with larger differences may have been selectively reported, but the effect of heterogeneity cannot be ruled out, raising the possibility of the existence of publication bias in published animal/iPSC models papers.

## 4 Discussion

For many years, studies into CF have relied on mouse models. In fact, the first genetically modified mouse models of CF disease were developed in 1994 [36]. However, it was clear that mice can exhibit human disease in different ways [37, 38]. As a result, researchers have started to use additional animal models that have similar CF pathological outcomes to humans [5], including pigs [39], and ferrets [40]. As all these animal models are well-characterized, our meta-analysis included studies that used them to generate mutant CF cells and were published between 2005 till 2018. Since the first generation iPSCs from human skin in 2007 [41], the iPSC technology has become a powerful and widespread laboratory technique for obtaining pluripotent stem cells from healthy individuals and patients with specific (genetic) diseases [42]. The use of human iPSCs has taken off with studies that investigated the ability of iPSCs to model diseases and allow for therapeutic screening applications [43]. In 2012, Wong A.P. and colleagues were the first to differentiate human pluripotent stem cells into mature airway epithelia expressing functional CFTR protein [44]. Our meta-analysis included studies that used iPSCs to generate mutant CF cells and were published between 2015 and 2020.

In this study, we presented a comparative summary of CFTR chloride channel function in animal and iPSC models. Our results revealed some issues with the quality of the studies included, primarily due to a lack of randomization and blinding, which is probably not feasible due to the type of investigation and the small sample size, to begin with. The sample sizes across studies were small meaning that there was inadequate control of random error partially contributing to the heterogeneity we have demonstrated. The results and interpretation of animal studies are not always straightforward because no single study is performed flawlessly in all steps; there is always a high possibility of bias from a variety of potential sources [45].

The I^2^ statistic revealed a great deal of inconsistency across studies in both animal and iPSC models. This means that the electrophysiological assessment of CFTR chloride channel function depends on the variability of both the procedure and tissue sensitivity across centers. Although both models had the same variability, iPSCs have a higher capacity to accurately and precisely model diseases because they are generated from the patient’s own cells, as opposed to animal models, which differ in how they present disease pathophysiology. Indeed, our meta-analysis revealed that animal models are incapable of reproducing CF pathology completely and accurately. While iPSC showed high heterogeneity, this could be attributed to the small number of studies that have used this model, limiting the certainty of statistical results interpretation. Therefore, such results need to be confirmed through further studies. Furthermore, the current state of our knowledge indicates that both animal and iPSC models are unlikely to produce reliable and reproducible results with precision unless the treatment has a large effect. These results are consistent with reports in the literature. For instance, a study compared the effects of omega-3 fatty acid supplementation and a control treatment amongst eight different animal models and found substantially heterogeneous results (I^2^ = 73%) [45]. In our study, similar results were found, and despite subgrouping studies by animal type, tissue type, ligand type and concentrations of ligand used in the animal model studies, there was still substantial heterogeneity. Our results as well as reports by others indicate that animals cannot be entirely relied on to model CF respiratory pathology.

The animal type was not a significant source of heterogeneity because all animal subgroups demonstrated significant heterogeneity. Only one study used ferrets, thus we could not analyze its contribution to heterogeneity. However, mice and pigs had an I^2^ value of 99.5% after the subgroup analysis. While we must be cautious in drawing conclusions based on I^2^ values because they may be skewed due to the small number of studies included, it is possible to conclude that using the same animal type to model CF respiratory pathologies is insufficient to achieve homogeneous results. This heterogeneity has also been witnessed in the literature repeatedly; variation present not only from one animal to the other, but even their individual pulmonary lobes showed significant variability in CF respiratory pathology modeling [46]. In line with this, Hooijmans and colleagues explain that individual biological differences between animals such as age and sex can lead to such heterogeneity, usually when data is not available to allow subgroup analysis [45]. For instance, male and female mice model CF differently regarding smooth muscle response, which plays a crucial role in asthma presentation of CF patients [47]. Also, ageing animals start demonstrating tissue remodeling [48]. Furthermore, mice model CF in a limited way because they do not exhibit severe respiratory pathology such as mucus plugging [49], which could be due to compensation for the defective chloride transport achieved by upregulation of the secretory pathway of the calcium-activated chloride channel present in the airway epithelia [50], in addition to not developing airway infections [37]. Another reason why mice cannot model human CF pathology is that they lack the characteristic cAMP-mediated chloride secretions reduction, besides other electrical defects [51]. Specifically, CFTR knockout mice models have shown the ability to overcome the insufficient and dysfunctional CFTR by upregulating the calcium-activated chloride channels [52].

In addition to mice, pigs have also been proven to model CF inconsistently, showing variable airway remodeling that includes goblet cell hyperplasia [53]. Moreover, newborn pigs lack the inflammation present in newborn babies [53], as compared to mice which can show excessive inflammation [37]. Additionally, one study looking at the pathogenesis of CF in pigs found that the lack of CFTR channels is associated with congenital sinus abnormalities resulting in 5 out of 9 pigs developing sinus disease [34]. Moreover, the 4 pigs that did not develop sinusitis developed lung disease or systemic illnesses. Interestingly, another study using murine models reported that CF rats did not develop spontaneous sinus disease like CF porcine models [32]. This demonstrates that animal model CF pathologies differ in presentation from not only each other but also from human CF pathologies. Perhaps it is because of these differences between animals that our analysis revealed such a high level of heterogeneity, even after subgroup analysis. This highlights the main limitation of animal models: their inability to consistently model human CF pathology.

The evidence in this study suggests that it is no different for iPSC models. Our observation in this meta-analysis is that iPSCs do not show better modelling capabilities compared to animal models. The source of iPSCs is usually somatic cells extracted from humans and hence should ideally represent human CF more accurately. However, we found that very few numbers of studies have been published studying the respiratory pathology of CF using iPSCs. For this reason, we consequently screened up until January 2021 specifically for iPSC studies but still, no additional studies fit our criteria. We were also unable to find other meta-analyses on the use of iPSCs in the context of CF. This was a limitation, but we still report substantial heterogeneity in the effect size of our iPSC studies (99.6%, Fig 5) like animal studies. This heterogeneity could be also due to similar factors as demonstrated by animal studies, but we do not have enough data to be conclusive. This lack of iPSC studies has likewise been a limitation with other meta-analyses investigating iPSC in other diseases and was also associated with heterogeneity. For example, a study investigating iPSC with regard to spinal injuries was able to include only 8 studies and had an I^2^ of 95.8%, indicating substantial heterogeneity. Moreover, the iPSC studies we analyzed focused largely on the initial work of differentiating the cells into CF and non-CF cells. Further investigation on the properties of these cells and their electrophysiological function is still to be done extensively. Notably, however, partial restoration of the CFTR channel was evident in CF iPSC cells, as compared to wildtype and corrected CF iPSC, and was achieved in cells treated with lumacaftor (VX809) [29]. VX809 has been tested out on adult CF patients in a randomized-controlled trial, which revealed improved CFTR function in the sweat gland [54]. While this meta-analysis was unable to answer the question of why the iPSC model is inconsistent, it does highlight the critical need for more iPSC publications in this field.

Another reason why I^2^ values should be interpreted with caution when determining whether heterogeneity is true or due to the very limited data pool and referring to previous meta-analyses is publication bias. It was found amongst both animal models and iPSC parameters in our study (LFK index of 4.81 and 6.64, respectively) (Fig 6). Such bias can considerably and nonlinearly influence the heterogeneity of the results from a meta-analysis, and I^2^ values were considered by a study to be invalid if such a large bias is present [55]. Publication bias was also found in all major parameters in a previous meta-analysis conducted regarding the induction of pulmonary artery hypertension in different animals. Indeed, publication bias is quite common when it comes to meta-analyses of animal models experimental studies [56]. As for iPSC studies, it is reasonable to speculate that this is due to the very low number of those studies and the general lack of iPSC studies published regarding CF respiratory pathologies, rather than a specific decrease in the number of null iPSCs studies published. It is very possible that as the number of iPSCs studies increases, the bias results will improve when compared to animal studies.

To the best of our knowledge, our meta-analysis is the first to compare iPSC and animal studies reporting CFTR chloride channel function. We found in this meta-analysis a high level of heterogeneity among results from animal/iPSC models. This heterogeneity may explain their limited capability in modelling CF. Given the potential of iPSC-derived models for studying various diseases, we recommend that more effort be put into developing reproducible iPSC models, as they have potential if properly developed. In addition, we cannot exclude other innovative cellular approaches that have shown promising results in modeling the diseases such as CRC stem-like cells, dual SMAD inhibition derived primary epithelial basal cells, and organoids.

## Supporting information

S1 Checklist PRISMA 2020 main checklist. (PDF)
