# Correction: Assessing the consistency of iPSC and animal models in cystic fibrosis modelling: A meta-analysis

**DOI:** 10.1371/journal.pone.0299166

**Published:** 2024-02-14

**Authors:** Toqa Darwish, Azhar Al-Khulaifi, Menatalla Ali, Rana Mowafy, Abdelilah Arredouani, Suhail A. Doi, Mohamed M. Emara

There are errors in the Funding section. The correct Funding statement is: The author acknowledges Qatar National Library for funding the publication of the article.
